# *Pseudomonas parafulva* SAPEU-1 as a keystone modulator: reshaping citrus phyllosphere microbiome to suppress citrus canker

**DOI:** 10.3389/fpls.2026.1771742

**Published:** 2026-03-23

**Authors:** Suhail Asad, Peng Gu, Fuyu Jiang, Jie Liu, Mei Chen, Samantha C. Karunarathna, Muhammad Atiq, Muhammad Younas, Pinnaduwage Neelamanie Yapa, Xurundong Kan, Jianqiang Zhang

**Affiliations:** 1 School of Tea and Coffee, Pu’er University, Pu’er, Yunnan, China; 2 Yunnan International Union Laboratory for Quality Monitoring and Evaluation of Agricultural Products in China and Malaysia, Pu’er, Yunnan, China; 3 School of Biology and Chemistry, Pu’er University, Pu’er, Yunnan, China; 4 Center for Yunnan Plateau Biological Resources Protection and Utilization & Yunnan International Joint Laboratory of Fungal Sustainable Utilization in South and Southeast Asia, College of Biology and Food Engineering, Qujing Normal University, Qujing, China; 5 Department of Plant Pathology, University of Agriculture, Faisalabad, Pakistan; 6 State Key Laboratory of Agricultural and Forestry Biosecurity, College of Plant Protection, Fujian Agriculture and Forestry University, Fuzhou, China; 7 Department of Biological Sciences, Faculty of Applied Sciences, Rajarata University of Sri Lanka, Mihintale, Sri Lanka

**Keywords:** biological control, endophytic bacteria, microbiome engineering, plant pathogens, plant–microbe interactions

## Abstract

**Introduction:**

Citrus canker disease, caused by the pathogen *Xanthomonas citri* subsp. *citri* (*Xcc*) poses a substantial challenge for citrus production due to the limited efficacy of chemical control and increasing pathogen resistance.

**Methods:**

In this study, we isolated an endophytic bacterial strain, Endophyte S2, from the phyllosphere of citrus plants in Yunnan Province, China. We evaluated its efficacy both as a biocontrol agent and as a modulator of the citrus leaf microbiome.

**Results and Discussion:**

*In vitro* antagonism assays revealed that Endophyte S2 achieved the highest inhibition rate (68.2%) against *Xcc* among all tested isolates. Molecular identification based on 16S rRNA gene sequencing classified S2 as *Pseudomonas parafulva* SAPEU-01. In greenhouse trials, citrus plants with *Xcc* infestation were treated with SAPEU-01, and phyllosphere samples were collected before treatment and one month after, and analyzed by Illumina MiSeq sequencing. Post-treatment, α-diversity (richness and evenness) increased significantly, and β-diversity (PCoA, Bray–Curtis) showed a clear separation of microbial community structure, with reduced intra-group variability. Taxonomic shifts included the enrichment of Proteobacteria (particularly Pseudomonadaceae and Sphingomonadaceae), as well as genera such as *Pseudomonas*, Sphingomonas, and Methylobacterium, concomitant with a marked decline in Xanthomonadaceae (including *X. citri*) and opportunistic taxa such as *Escherichia coli* O157:H7 and *Klebsiella aerogenes*. Beneficial taxa, including *Leuconostoc tardus*, *Sphingomonas*, and *Curtobacterium luteum*, also increased. These results suggest *Pseudomonas parafulva* SAPEU-01 not only suppresses the pathogen but also restructures the phyllosphere microbiome toward greater stability and potential resilience.

## 1 Introduction

Citrus is among the world’s most economically important fruit crops in tropical and subtropical regions. The crop is cultivated in more than 130 countries worldwide but faces a severe limitations due to multiple biotic and abiotic stresses. However, citrus canker, caused by *Xanthomonas citri* subsp. *citri* (*Xcc*) bacteria are the primary threat to the successful cultivation and cause heavy economic losses. This disease results in erumpent lesions appearing on leaves, stems, and fruits, leading to complete defoliation and plant death (Gottwald et al., 2002). The current control measures include extensive spraying with copper solutions and antibiotics, and the removal of infected trees. However, such strategies raise multiple environmental concerns, and injudicious use further enhances the likelihood of resistance development (Behlau et al., 2021; Batuman et al., 2024).

Research advances in plant microbiome studies have identified host microbiome manipulation as a novel strategy for plant protection. Such strategies primarily employ the phyllosphere, which harbors a dynamic microbiome and further regulates host health and suppresses pathogens (Vorholt, 2012; De Mandal and Jeon, 2023). Although phyllosphere microbiome studies have remained underexplored compared to rhizosphere microbiome studies, growing evidence indicates that leaf microbiota contributes to resistance against leaf pathogens (Stone et al., 2018; Sivakumar et al., 2020). The entry and accumulation of pathogens trigger disease and disrupt the microbiome’s community structure and composition (Li et al., 2022). Similarly, citrus canker has been identified as accelerating inter-kingdom interactions within the microbiota and inducing a disorder in the leaf microbiota (Huang et al., 2023).

Endophytic bacteria in the phyllosphere microbiome are particularly promising for biological control due to their relative immunity to external stresses (UV and dehydration) and their potential to interact closely with the host at a physiological level. Specific investigations have identified leaf microbiota with antagonistic potential against phytopathogens in citrus and other crops (Daungfu et al., 2019; Rabbee et al., 2022). Most such candidates have not been able to establish themselves successfully in the field, and their performance is often inconsistent under commercial conditions (Emmert and Handelsman, 1999; May et al., 2021).

*Pseudomonas* is a genus with high potential for biological control. It has been shown to produce a large number of antimicrobial substances (e.g., phenazines and lipopeptides), secrete hydrolytic enzymes, outcompete for limited available nutrients (e.g., siderophore production), and induce host-defense reactions (Gross and Loper, 2009; Legein et al., 2020). Additionally, it has been considered as “green guardian” with high applicability to sustainable plant disease control methods, particularly due to its metabolic flexibility and competitiveness (Yang et al., 2025). Specifically, *Pseudomonas parafulva* genome analyses have identified genes for phenazine biosynthesis, including phenazine-1-carboxylic acid (PCA), which directly contributes to antimicrobial activity (Zhang et al., 2019). Furthermore, antagonism, motility, and siderophore production were identified to be crucial for the biological control capacity of *Pseudomonas parafulva* JBCS1880 against bacterial diseases in soybean crops (Kakembo and Lee, 2019). The majority of biocontrol research has focused on two main areas which include pathogen suppression and plant growth promotion but scientists have studied these methods less for their effects on natural microbial communities found in local ecosystems (Rashi Gupta et al., 2014; Berg et al., 2021). Few studies have analyzed *in vitro* inhibition tests, phylogenetic identification, and community-level analyses of alpha and beta diversity or other functions, given the increasing acceptance of the importance of multiple levels of analysis in biocontrol studies (Mitter et al., 2016). Moreover, the concept of using a biocontrol inoculant to actively restore or redirect community structure, rather than merely eliminating pathogens, remains underexplored. The long-term field stability of such induced community states is hardly tested (Trivedi et al., 2020).

To address these gaps, an integrated pipeline was developed: (1) isolate indigenous endophytic bacteria from citrus phyllosphere in Yunnan Province, China; (2) screen for antagonistic activity against *Xcc* via dual culture assays; (3) identify the most potent strain by 16S rRNA sequencing and phylogenetic analysis; (4) inoculate canker-infected citrus under controlled pot conditions; and (5) perform high-throughput Illumina sequencing of phyllosphere bacterial communities (before vs. after treatment), analyzing α- and β-diversity, taxonomic shifts at multiple ranks, co-occurrence network restructuring, and predicted functional changes. The hypothesis is that a well-selected indigenous endophyte can not only suppress the pathogen, but also act as a keystone modulator to recalibrate the native microbiome—enhancing richness, stability, and beneficial taxa. This synergistic approach bridges microbial ecology, phylogenomics, biocontrol applications, and functional inference.

## 2 Materials and methods

### 2.1 Collection of samples

Leaf samples were collected to isolate the indigenous bacterial endophytes from the citrus growing areas (Baoshan, Dali, Dehong, Lincang, Xishuangbanna) of Yunnan Province, People’s Republic of China. Yunnan Province (21–29° N, 97–106° E) is characterized by a highly heterogeneous landscape (76–6,740 m a.s.l.) and a monsoon climate, receiving 600–2,300 mm of annual rainfall, predominantly during the wet season (**Table 1**) (Gu et al., 2016; Zhang et al., 2025; Li et al., 2025; He et al., 2014). The samples were collected in polyethene bags, immediately transferred to an ice box, and then brought to the laboratory for the isolation and purification of bacterial endophytes.

**Table 1:** Geographic characteristics of citrus leaf sampling sites in Yunnan Province, China.

Region	Latitude (N)	Longitude (E)	Approx. elevation (m)	Description
Baoshan	~25°07′	~99°10′	1,530–2,640	Subtropical highland; mountainous agricultural region
Dali	25°25′–25°58′	99°58′–100°27′	~1,970	Low-latitude plateau monsoon climate; intensive agriculture
Dehong	23°50′–25°20′	97°31′–98°43′	~1,562 (500–3,503)	Subtropical mountainous border region
Lincang	23°05′–25°03′	98°40′–100°32′	~1,650 (450–3,504)	Subtropical monsoon climate; predominantly mountainous
Xishuangbanna	21°08′–22°36′	99°56′–101°51′	~1,000 (370–2,429)	Tropical monsoon climate; highly biodiverse mountainous region

### 2.2 Isolation and purification of bacterial endophytes

The healthy citrus leaf samples were washed with tap water, and the surfaces were sterilized in 70% ethanol for 30 s, followed by 2% sodium hypochlorite for 1 min, and then rinsed in sterile water. Following surface sterilization, the leaf tissues were aseptically macerated using a sterile mortar and pestle in sterile distilled water to obtain a homogenized suspension. The homogenate was then serially diluted (10^-1^–10^-4^) and 100 µL aliquots were spread on LB agar from each dilution and incubated at 30 to 37 °C for 48 h. After that, morphologically distinct colonies were selected and subcultured three times by streaking to ensure purity. Purified isolates were further stored in glycerol (50%) at –80 °C for long-term use (Ke et al., 2023).

### 2.3 The antagonistic approach of bacterial endophytes against the *Xanthomonas citri* subsp. *citri*

Antagonistic activity of the endophytic bacterial isolates against *Xanthomonas citri* subsp. *citri* was evaluated using an agar diffusion assay. A 200 µL suspension of the pathogen (10^-1^ dilution) was evenly spread onto LB agar plates to form a uniform bacterial lawn. After allowing the surface to dry (~15 min), 2 µL aliquots of each endophytic isolate were spot-inoculated onto the plates. The plates were then incubated at 28 °C for 48 h, and inhibition zones were assessed to determine antibacterial activity and growth inhibition ratios were calculated as [(Inhibition zone−Colony diameter)/Inhibition zone]×100% following established protocols (Kjeldgaard et al., 2022).

### 2.4 DNA extraction

The bacteria were grown until the mid-to-late log phase (0.5-0.7 at OD600), and 1 mL of the culture was centrifuged at 7,500 rpm for 10 minutes. DNA was extracted using the cetyltrimethylammonium bromide (CTAB) method with slight modifications (Araújo et al., 2002). The pellet was resuspended in Tris-EDTA (TE) buffer, and 525 µL PCI (Phenol:chloroform: Isoamyl) solution was added to the tube, followed by centrifugation at 12,000 rpm for 15 minutes. An equal volume of chilled isopropanol was added to the resulting supernatant, and the mixture was again centrifuged as above. The pellet was resuspended in 50 µL of distilled water and incubated overnight at 4 °C. The presence and concentration of bacterial DNA were confirmed by running 5 µL of product on a 1.5% agarose gel. Purified DNA will appear as a defined band when visualized under UV light.

### 2.5 Molecular characterization using amplification of the 16S rRNA gene

Molecular identification of the selected endophytic isolates was conducted by amplifying nearly full-length 16S rRNA genes using universal primers 27F (5′-AGAGTTTGATCCTGGCTCAG-3′) and 1492R (5′-TACGGYTACCTTGTTACGACTT-3′) under the following PCR conditions: initial denaturation at 94 °C for 4.5 min, followed by 30 cycles of denaturation at 94 °C for 40 s, annealing at 55 °C for 30 s, extension at 72 °C for 1 min, and a final extension at 72 °C for 10 min. Amplicons were purified, cloned into the PCR^®^-TOP10 vector (Tiangen Biotech), and sequenced. Subsequent sequence alignment and phylogenetic reconstructions confirmed the taxonomic affiliation (Frank et al., 2008; Dos Santos et al., 2019).

### 2.6 Sequencing and phylogenetic analysis

The gene sequences of the selected endophytic bacterial isolates and relevant reference taxa were retrieved from the NCBI database (Accession Number: PQ895797). Multiple sequence alignment was performed using ClustalW implemented in MEGA version 7.0. The alignment was manually inspected and trimmed to remove poorly aligned regions. A phylogenetic tree was constructed using the Neighbor-Joining method with the Kimura two-parameter (K2P) model, and the robustness of the tree topology was evaluated using 1,000 bootstrap replicates. The resulting tree was visualized using MEGA to infer the evolutionary placement of the isolate (Tamura et al., 2011).

### 2.7 Microbial diversity analysis

#### 2.7.1 Plant material and experimental design

The experiment was conducted on three biological replicates of diseased citrus plants inoculated with the selected strain Endophyte *Pseudomonas parafulva* SAPEU-01(10^8^ CFU mL^-1^) and evaluated “before” (day 0) and “after” treatment (day 30) to assess microbial community shifts and disease suppression, in accordance with practices in early-stage greenhouse biocontrol trials.

#### 2.7.2 Sample collection

Leaves were collected before and after the application of *Pseudomonas parafulva* SAPEU-01, following three replicates. Leaves were transferred to polythene bags, stored at -80 °C, and then sent to the laboratory for further analysis.

### 2.8 DNA extraction and PCR amplification

Total microbial community DNA was extracted from surface-sterilized citrus leaf tissues by Majorbio Bio-Pharm Technology Co., Ltd. (Shanghai, China) following standardized protocols. The microbial community genomic DNA extract was checked on a 1% agarose gel, and DNA concentration and purity were determined using a NanoDrop 2000 UV-vis spectrophotometer (Thermo Scientific, Wilmington, USA). The hypervariable region V3-V4 of the bacterial 16S rRNA gene was amplified with primer pairs 338F (5’-ACTCCTACGGGAGGCAGCAG-3’) and 806R(5’-GGACTACHVGGGTWTCTAAT-3’) by an ABI GeneAmp^®^ 9700 PCR thermocycler (ABI, CA, USA). The PCR amplification of the 16S rRNA gene was performed as follows: initial denaturation at 95 °C for 3 min, followed by 27 cycles of denaturing at 95 °C for 30 s, annealing at 55 °C for 30 s, extension at 72 °C for 45 s, and single extension at 72 °C for 10 min, and ended at 4 °C. The PCR mixtures contain 5 × *TransStart* FastPfu buffer (4 μL), 2.5 mM dNTPs (2 μL), forward primer (5 μM) (0.8 μL), reverse primer (5 μM) (0.8 μL), TransStart FastPfu DNA Polymerase (0.4 μL), template DNA (10 ng), and finally ddH_2_O up to 20 μL. PCR reactions were performed in triplicate. The PCR product was extracted from a 2% agarose gel and purified using the AxyPrep DNA Gel Extraction Kit (Axygen Biosciences, Union City, CA, USA) according to the manufacturer’s instructions, and then quantified using a Quantus Fluorometer (Promega, USA).

### 2.9 Illumina MiSeq sequencing

Purified amplicons were pooled in equimolar amounts and paired-end sequenced on an Illumina MiSeq PE300 platform/NovaSeq PE250 platform (Illumina, San Diego, USA) according to standard protocols by Majorbio Bio-Pharm Technology Co., Ltd. (Shanghai, China). The raw reads were deposited into the NCBI Sequence Read Archive (SRA) database (Accession Number: PRJNA1214773).

### 2.10 Processing of sequencing data

The raw 16S rRNA gene sequencing reads were demultiplexed, quality-filtered by fastp version 0.20.0 (Chen et al., 2018) and merged by FLASH version 1.2.7 (Magoč and Salzberg, 2011), with the following criteria: (i) the 300 bp reads were truncated at any site receiving an average quality score of <20 over a 50 bp sliding window, and the truncated reads shorter than 50 bp were discarded, reads containing ambiguous characters were also discarded; (ii) only overlapping sequences longer than 10 bp were assembled according to their overlapped sequence. The maximum mismatch ratio of the overlap region is 0.2. Reads that could not be assembled were discarded; (iii) Samples were distinguished according to the barcode and primers, and the sequence direction was adjusted, with exact barcode matching and two nucleotide mismatches in primer matching. Operational taxonomic units (OTUs) with a 97% similarity cutoff were clustered using UPARSE version 7.1 (Edgar, 2013; Stackebrandt and Goebel, 1994), and chimeric sequences were identified and removed. The taxonomy of each OTU representative sequence was analyzed by RDP Classifier version 2.2 against the 16S rRNA database (e.g., Silva v138) using a confidence threshold of 0.7 (Wang et al., 2007).

### 2.11 Statistical analysis

Data were statistically analyzed using a t-test (P < 0.05). All statistical analysis was performed using IBM SPSS Version 20.0 (SPSS Inc., Chicago, IL, USA). QIIME software (Version 1.9.1) calculates the Observed-OTUs, Chao1, Shannon, and ACE indices. The Bray-Curtis dissimilarity matrix was calculated for beta-diversity analysis of bacterial and fungal communities and used for principal coordinate analysis (PCoA) in QIIME. All figures were processed and illustrated using Adobe Illustrator CC 2025 (Adobe Systems Inc., San Francisco, CA, USA).

## 3 Results

### 3.1 *In vitro* antagonism of endophytic isolates against *Xanthomonas citri* subsp. *citri*

**Figure 1:**
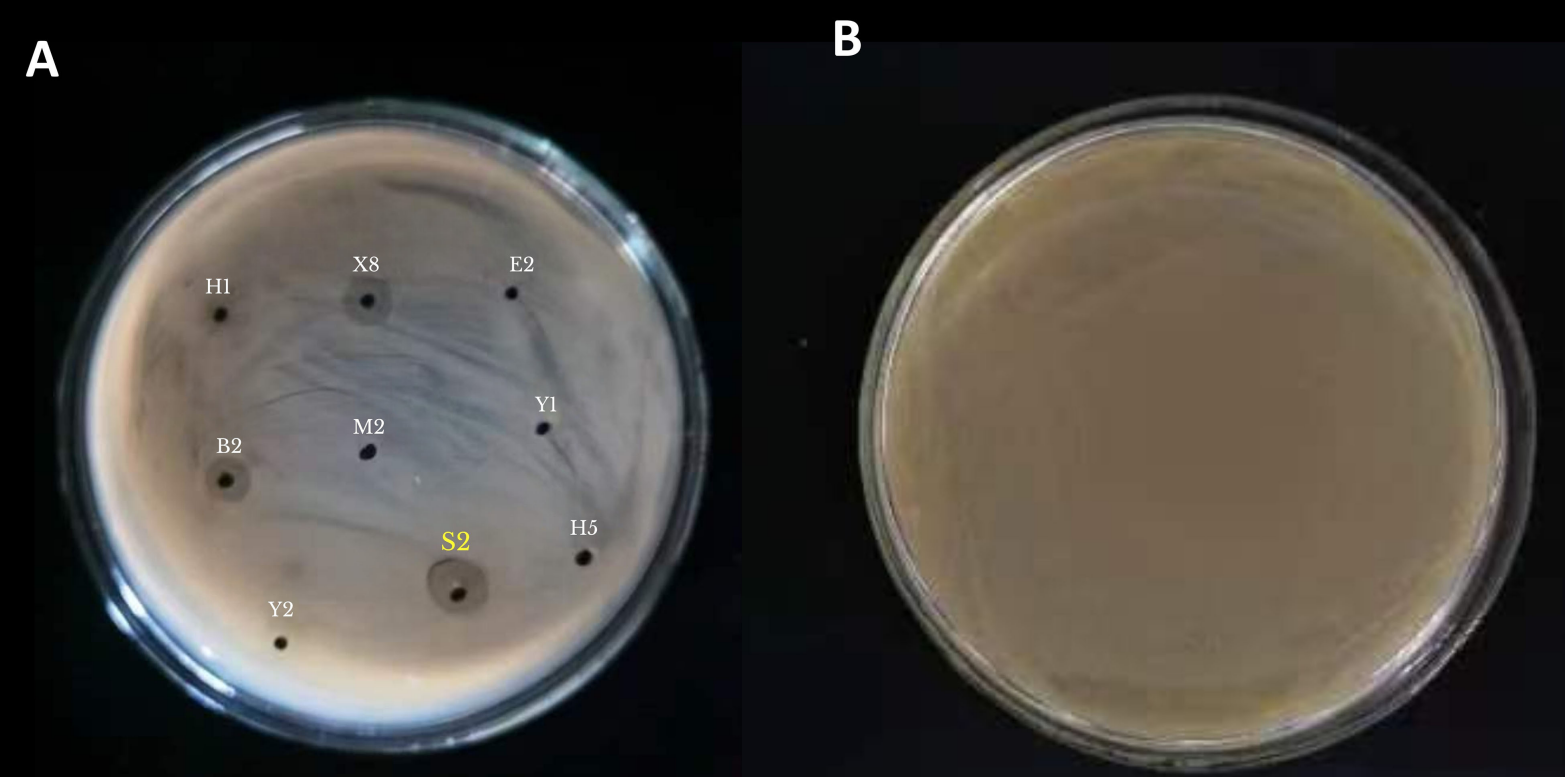
*In vitro* antagonistic activity of endophytic bacterial strains against *Xanthomonas citri* subsp. *citri*. **(A)** Inhibition zones produced by different endophytic isolates on LB agar plates. **(B)** Control (CK): pathogen growth without endophytic bacteria.

The *in vitro* dual-culture antagonism testing demonstrated significant variability among the 12 evaluated endophytic strains in their ability to suppress *Xcc*. The study found that strain S2 had the highest antibacterial efficacy against *Xcc*, with an inhibition ratio of 68.2% (**Figure 1**). Strain X8 also showed robust performance, suggesting overlapping antagonistic efficacy with S2. Intermediate antagonists, such as B2, H1, X3, and E2, exhibited lower inhibition than S2 and X8, but were significantly greater than the weakest strains. S2 emerged as the most formidable opponent to *Xcc* (**Table 2**).

**Table 2:** Antagonistic activity of endophytic bacterial isolates against *Xanthomonas citri* subsp. *citri*.

Bacterial species	Inhibition zone (mm)	Colony diameter (mm)	Growth inhibition ratio (%)
B2	18.66 ± 0.15	11.33 ± 0.15	39.3 ± 0.7 b
H1	17.66 ± 0.15	11.33 ± 0.15	35.9 ± 1.4 b
Y2	15.33 ± 0.15	11.33 ± 0.15	26.1 ± 1.5 c
P2	12.66 ± 0.15	9.66 ± 0.15	23.7 ± 1.9 c
X3	15.33 ± 0.15	10.33 ± 0.15	32.6 ± 1.4 b
S2	28.33 ± 0.15	9.00 ± 0.26	68.2 ± 0.9 a
H5	14.66 ± 0.15	11.33 ± 0.15	22.7 ± 1.4 c
M2	15.33 ± 0.15	11.33 ± 0.15	26.1 ± 1.5 c
Y1	12.66 ± 0.15	9.66 ± 0.15	23.7 ± 1.9 c
E2	15.33 ± 0.15	10.33 ± 0.15	32.6 ± 1.4 b
X8	24.33 ± 0.15	9.00 ± 0.26	63.0 ± 0.9 ab

Values are means ± standard error (SE). Growth inhibition ratio is calculated as (Inhibition zone diameter−Colony diameter)/Inhibition zone diameter×100%. Different lowercase letters (a, b, c) following means indicate statistically significant differences (p < 0.05) by *post hoc* multiple comparison test (e.g., Tukey’s HSD).

### 3.2 Phylogenetic identification of the antagonistic endophyte

**Figure 2:**
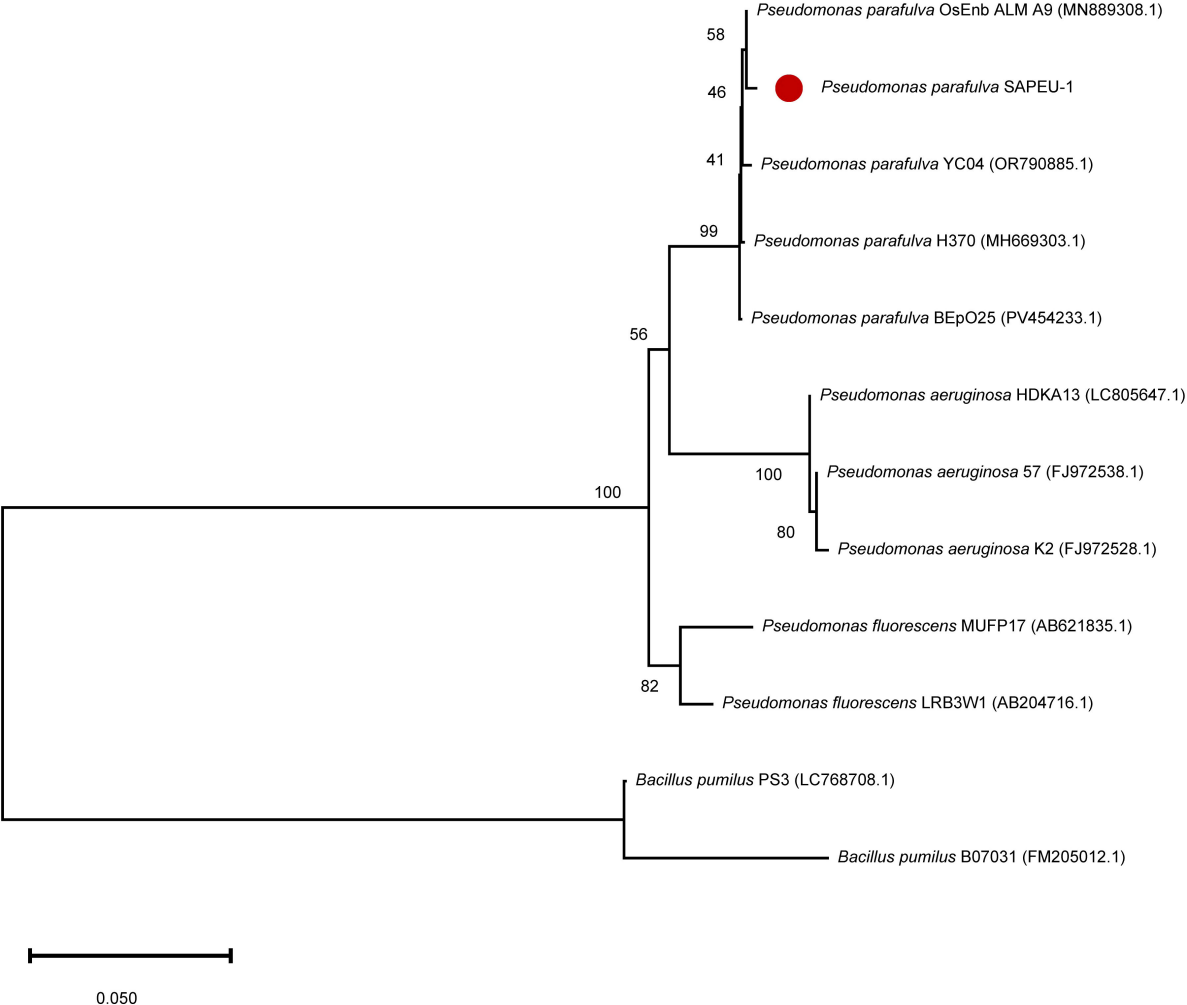
Neighbor-joining phylogenetic tree based on partial 16S rRNA gene sequences showing the taxonomic position of the antagonistic endophyte *Pseudomonas parafulva* SAPEU-1 (red dot). Bootstrap values (greater than 40%) based on 1,000 replicates are shown at branch nodes. The scale bar represents 0.05 substitutions per nucleotide position. *Bacillus pumilus* sequences were used as an outgroup.

A neighbor-joining phylogenetic tree based on the 16S rRNA gene of the potent antagonistic isolate S2 indicated that the strain *Pseudomonas parafulva* SAPEU-1 (*P. parafulva* SAPEU-1), a potent antagonistic isolate, is closely related to several *P. parafulva* reference sequences, including *P. parafulva* OsEnb ALM A9, YC04, H370, and BEPo25. Moderate bootstrap values support this grouping, confirming its close evolutionary relatedness within the *P. parafulva* clade. This cluster is clearly separated from *P. aeruginosa* and *P. fluorescens*, which form distinct branches. More distantly related taxa, such as *Bacillus pumilus* strains PS3 and B07031, serve as outgroups, reinforcing the phylogenetic distinction of the *Pseudomonas* lineage. The endophytic isolate S2 belongs to *P. parafulva*, confirming its placement within the genus *Pseudomonas* but distinct from the pathogenic *P. aeruginosa* complex (**Figure 2**).

### 3.3 Interpretation of α-diversity responses to *P. parafulva* SAPEU-1 treatment

**Figure 3:**
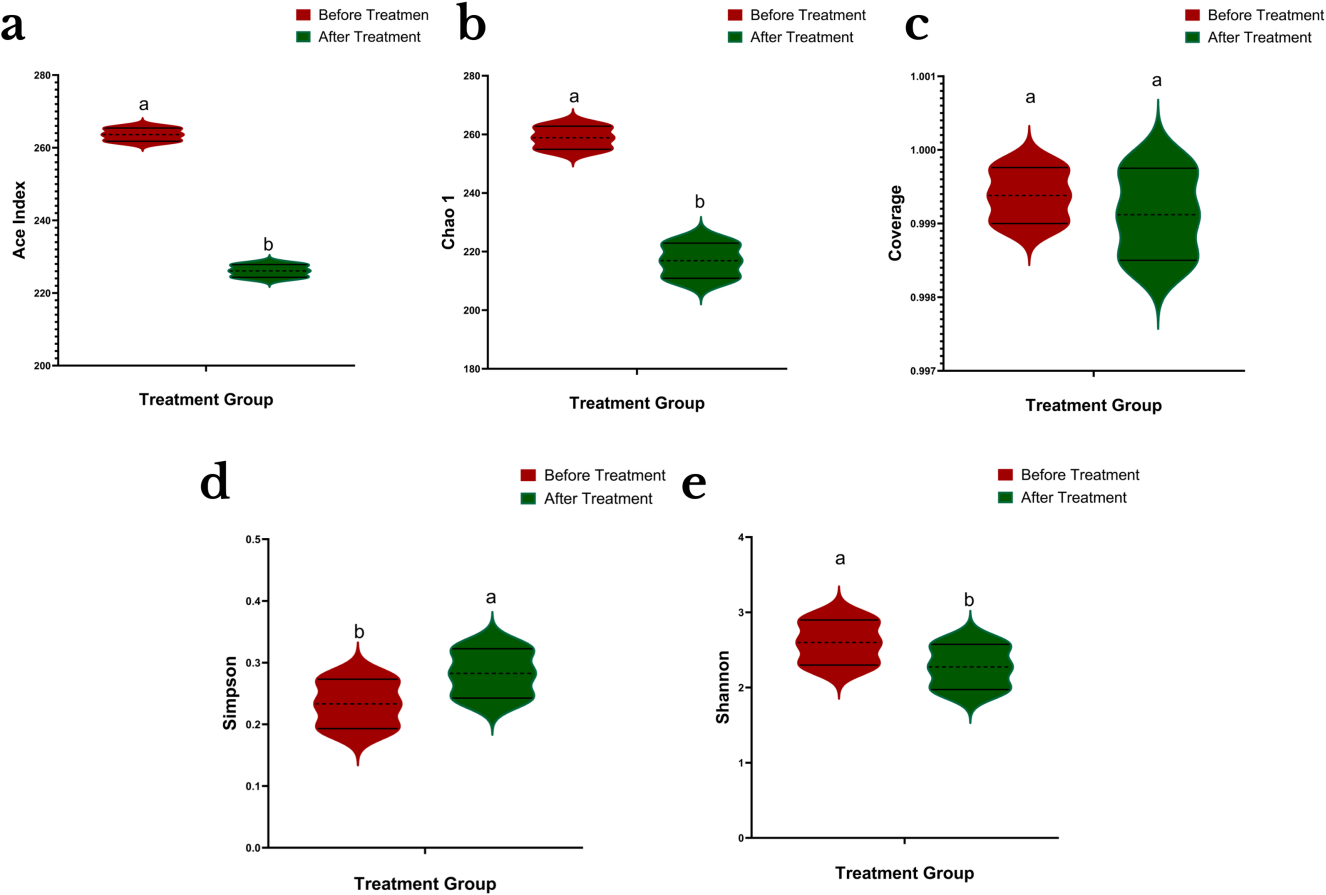
Comparison of α-diversity indices in citrus phyllosphere communities before and after treatment with *Pseudomonas parafulva* SAPEU-1. Violin plots show the distribution of **(a)** ACE index, **(b)** Chao1 richness estimator, **(c)** Coverage, **(d)** Simpson index, and **(e)** Shannon diversity index for samples collected before (red) and after (green) treatment. Letters above violin plots indicate statistically significant differences between groups (p < 0.05, ANOVA/Tukey’s test).

The alpha-diversity study showed that *P. parafulva* SAPEU-1 inoculation of citrus canker leaves resulted in significant changes to bacterial community patterns at two different time points. The value of diversity in the treated plants always remained greater than that of the untreated ones. The Ace and Chao1 indices showed that rare and low-frequency phyllosphere microbiota numbers rose substantially during the post-treatment assessment. Shannon and Simpson indices showed both increases and decreases in their post-treatment values, suggesting an increase in phyllosphere microbiota richness and a relatively even distribution of bacteria. The Coverage index reached 1.0 for both samples, which showed the depth of sequencing and the precision of microbial community documentation. The *P. parafulva* SAPEU-1 strain enhanced both leaf microbial diversity and homogeneity in citrus plants suffering from citrus canker disease, which indicates it helped preserve the natural balance of phyllosphere microbial communities (**Figure 3**).

#### 3.3.1 Shared and unique OTU composition before and after treatment

Results revealed that a transformation in community structure occurs following inoculation of *P. parafulva* SAPEU-1 into citrus leaf phyllosphere communities. The pre-treatment community comprised 567 OTUs, of which 44.51% were unique to this community, and 170 OTUs were shared between both communities. In the post-treatment community, 495 OTUs were identified, of which 36.43% were unique to the post-treatment state. Even with 19.06% OTUs being common to both treatment and post-treatment communities, there is a large percentage of OTUs unique to each community. This highlights the high community turnover post-treatment. The total number of OTUs did not decline drastically, with a net loss of 72 OTUs, leaving it slightly lower at 495.

Nevertheless, despite the overall reduction in total OTUs, a large number of novel OTUs were identified post-treatment that had not been previously identified or observed. This indicates recruitment or amplification of rare OTUs, or of those that could not be identified or monitored. This corresponds to 19.06% of OTU richness, which is recognized as common to both the treatment and post-treatment groups. This suggests a core community that remains consistent across both the treatment and post-treatment periods. This community could consist of OTUs that have not been affected or altered under either treatment or post-treatment. This again corresponds to OTUs that can thrive or survive in their current microenvironment under the selective pressure exerted by *P. parafulva* SAPEU-1.

### 3.4 β-diversity and community structure analysis

This data analysis employed Principal Coordinates Analysis (PCoA) to determine the structure of the citrus leaf bacterial assemblages before inoculation with *Pseudomonas parafulva* SAPEU-1. The total variance accounted for by the first two principal coordinates, PC1 and PC2, was 51.36% and 27.92%, respectively. These two parameters collectively accounted for approximately 79.3% of the total variance in overall community dissimilarities. The samples obtained before treatment remained in a tight cluster on the positive coordinate of PC1. The post-inoculation samples grouped uniquely on the negative side of the coordinate. This unique pattern of community distribution indicates that the *P. parafulva* SAPEU-1 inoculation treatment had a profound impact on the structure of the citrus leaf bacterial community. This is evident because it led to the establishment of a novel and relatively homogeneous community structure compared to the diseased control. The one-month change in community structure suggests that *P. parafulva* SAPEU-1 has exerted selective pressure on the indigenous microbiota. This action is likely to have suppressed the growth of pathogen-related bacteria. It also promoted novel or commensal bacteria.

#### 3.4.1 Community dissimilarity and compositional stability

A comparison of Bray-Curtis dissimilarities among and within sample groups showed that the composition of bacteria differed significantly between the pre- and post-inoculation periods with *P. parafulva* SAPEU-1. The between-group dissimilarities were larger than the within-group dissimilarities, which showed that the community is relatively homogeneous and stable. The post-treatment group showed the smallest Bray-Curtis dissimilarities, indicating that inoculation with *P. parafulva* SAPEU-1 resulted in a stable structure. The lower variability within the groups and larger between-group variance showed that *P. parafulva* SAPEU-1 treatment had influenced the phyllosphere microbiome community structure to form a stable post-treatment community structure. This indicated that inoculation with *P. parafulva* SAPEU-1resulted in increased microbial diversity and improved convergence.

#### 3.4.2 Comparison of richness (ace index) before and after treatment

**Figure 4:**
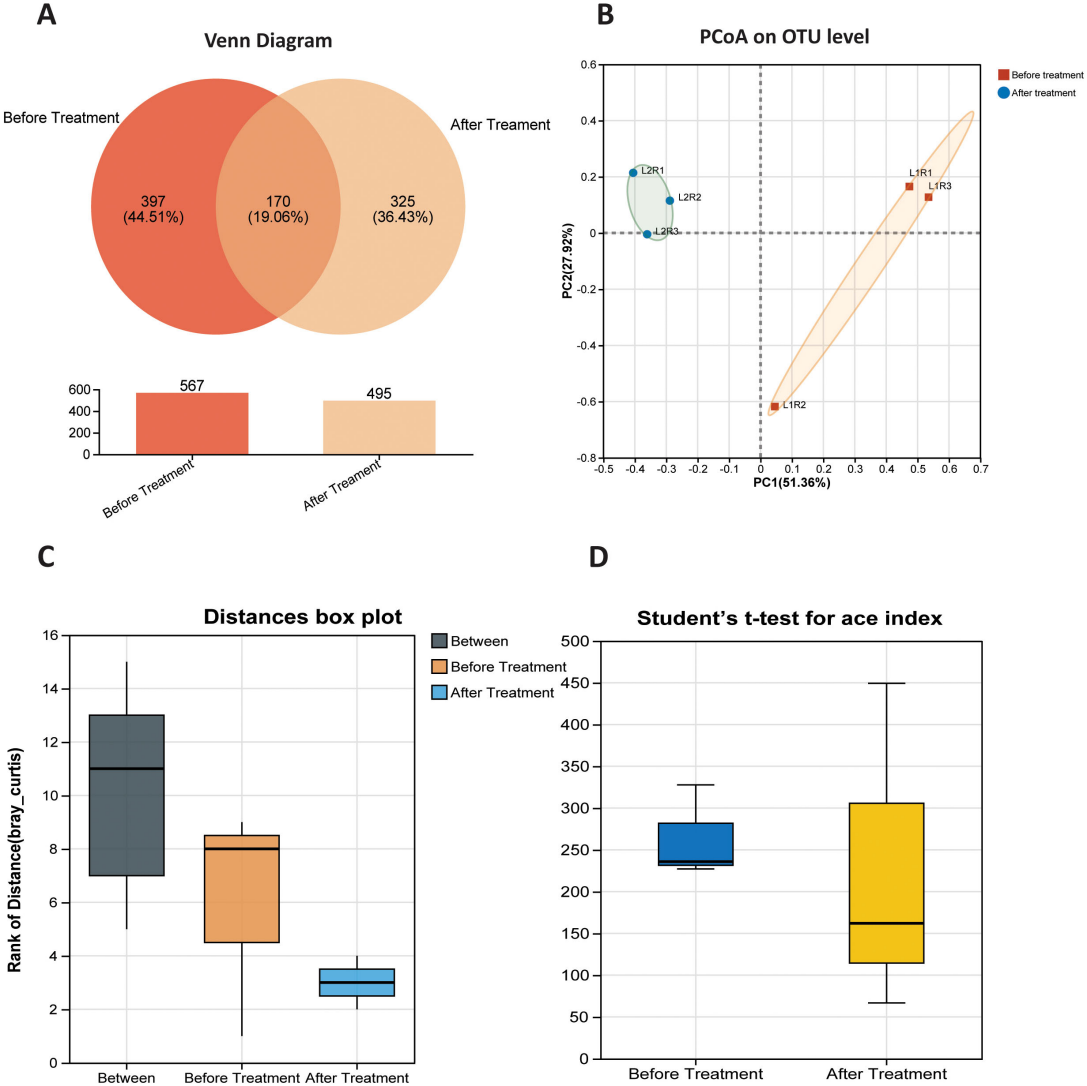
Changes in microbial community composition and diversity at the OTU level before and after treatment. **(A)** Venn diagram showing shared and unique OTUs between before- and after-treatment groups. **(B)** Principal coordinates analysis (PCoA) based on Bray–Curtis dissimilarity, illustrating differences in microbial community structure between treatments. Percentages on axes indicate the proportion of variance explained by each coordinate. **(C)** Bray–Curtis distance box plot comparing within-group (before and after treatment) and between-group community dissimilarities. **(D)** Box plot of the ACE richness index before and after treatment. Statistical differences were assessed using Student’s *t*-test.

The Ace diversity index was measured in citrus canker leaves before and after inoculation with *P. parafulva* SAPEU-1. The Ace index showed significantly values, in post-inoculation samples compared to pre-inoculation samples, indicating a reduction in microbial richness after treatment. These findings confirm that *P. parafulva* SAPEU-1 inoculation influenced the microbial community structure of the citrus leaf phyllosphere. These results suggest that *P. parafulva* SAPEU-1 inoculation contributed to restructuring and rebalancing the citrus leaf phyllosphere microbiota rather than increasing microbial richness (**Figure 4D**).

#### 3.4.3 Taxonomic composition of bacterial communities at the phylum level

Results showed that both samples were dominated by Proteobacteria, with a percentage of 66.20% before treatment and 73.45% post-treatment with *P. parafulva* SAPEU-1. A slight decrease was noted in Actinobacteriota and a small drop in Firmicutes. A low value was recorded for Bacteroidota, ranging from 0.43% to 1.21%. Suborders such as Armatimonadota, Myxococcota, and Chloroflexi represented small orders that showed little change across treatments. It is noted that the increase in Proteobacteria indicates the role of *P. parafulva* SAPEU-1. In ameliorating the growth of Proteobacteria species, thereby supporting disease and microbiota stabilization.

#### 3.4.4 Community composition at the family level

**Figure 5:**
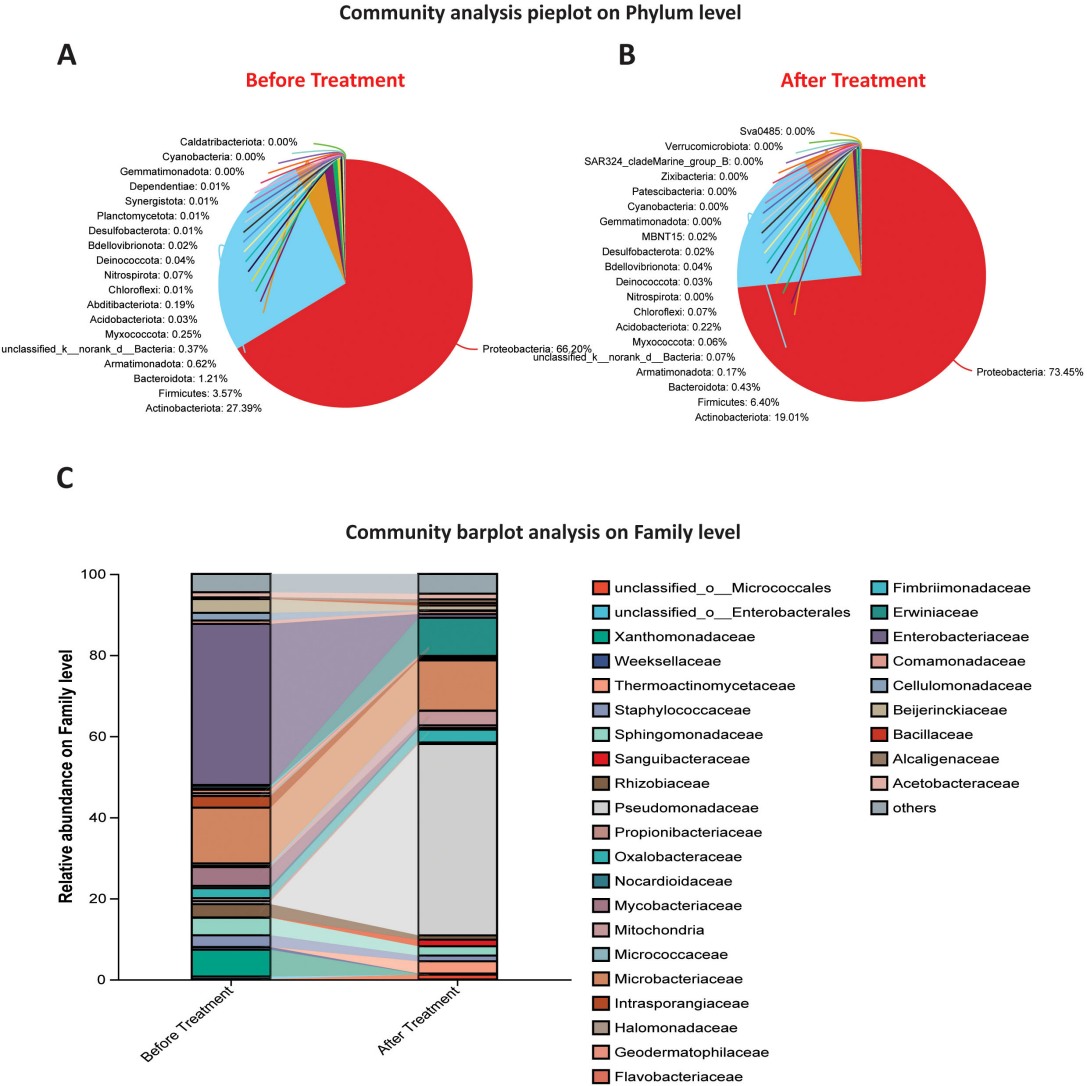
Community composition of bacterial communities before and after *Pseudomonas parafulva* SAPEU-01 treatment. **(A)** Phylum-level relative abundance in leaf samples before treatment (n = 3). **(B)** Phylum-level relative abundance in leaf samples after treatment (n = 3). **(C)** Bar-plot showing family-level relative abundances of key bacterial families before and after treatment; colors correspond to families listed in the legend.

The analysis at the family level elucidated compositional alterations caused by *P. parafulva*, introducing modifications to the Micrococcaceae, Nocardiaceae, Staphylococcaceae, and Xanthomonadaceae families of bacteria, which have been identified as associated with the pathogen causing citrus canker disease. After inoculation with *P. parafulva* SAPEU-1, the amount of Pseudomonadaceae and Sphingomonadaceae increased. At the same time, there was a decrease in the amount of Xanthomonadaceae and Staphylococcaceae. An increase in the amount of Comamonadaceae, Beijerinckiaceae, and Rhizobiaceae families can represent the successful colonization of *P. parafulva* SAPEU-1 in the citrus phyllosphere. This also suggests that *P. parafulva* SAPEU-1 inhibits or suppresses pathogenic or opportunistic bacteria associated with disease-causing microbes, such as those in the Xanthomonadaceae family (**Figure 5**).

#### 3.4.5 Genus-level composition and differential abundance analysis

##### 3.4.5.1 Circos plot of genus-level associations

**Figure 6:**
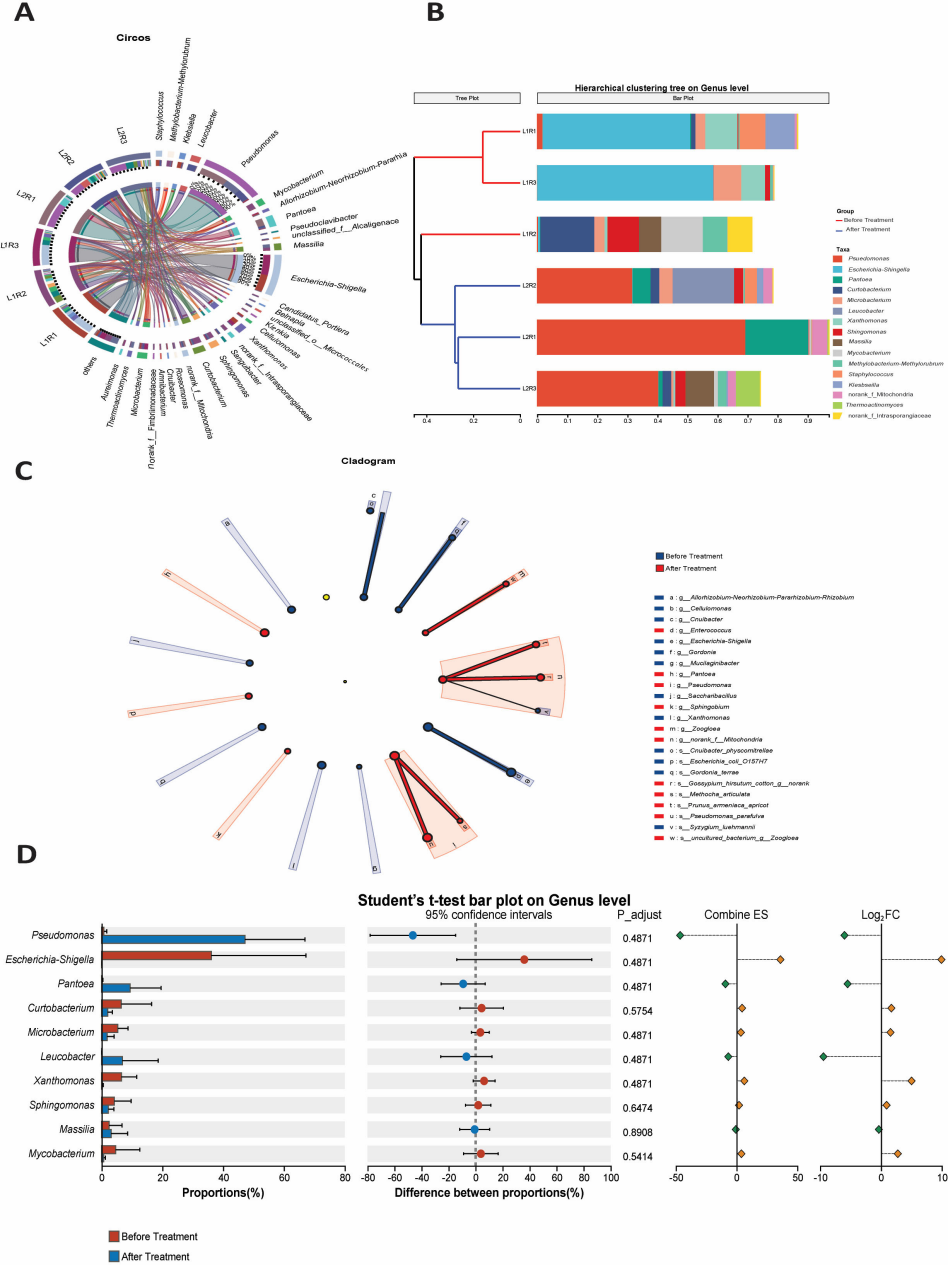
Genus-level bacterial community structure and differential abundance in citrus canker leaves before and after *Pseudomonas parafulva* SAPEU-01 treatment. **(A)** Circos plot illustrating genus-sample associations and relative abundances. **(B)** Hierarchical clustering of dominant genera showing separation between before- and after-treatment samples. **(C)** LEfSe cladogram identifying taxa enriched in each group. **(D)** Student’s *t*-test and log-fold-change analysis of major genera, highlighting the enrichment of *Pseudomonas* and suppression of *Xanthomonas* after *Pseudomonas parafulva* SAPEU-01 application.

The Circos diagram (**Figure 6A**) illustrates the relationships between various taxonomic groupings and their relative prevalence among prominent bacterial genera in leaves affected by citrus canker. This is done before and after inoculation with *P.parafulva* SAPEU-1. The connections between samples show the distribution of samples among the genera. The outer tracks of the plot reveal that *Pseudomonas* is the prevailing genus in the inoculated samples with high correlation to the *Pseudomonas*-inoculated replicates (L2R1-L2R3). For the non-inoculated infected samples (L1R1-L1R3), there is a high relative abundance of *Xanthomonas*, *Microbacterium*, and *Staphylococcus*. The variance in intensity between the non-inoculated and inoculated samples indicates significant disruption of the microbiome at the genus level following treatment with *P. parafulva* SAPEU-1.

##### 3.4.5.2 Hierarchical clustering of dominant genera

Hierarchical cluster analysis (**Figure 6B**) revealed that bacterial genera were grouped based on compositional similarities. The dendrogram revealed a distinct pattern between the pre- and post-treatment samples, indicating a significant disruption of community structure. The pre-treatment samples were mainly composed of genera associated with disease or potential opportunistic colonization, including *Xanthomonas*, *Microbacterium*, and *Staphylococcus*. On the contrary, the post-treatment samples showed high *levels of Pseudomonas, Sphingomonas, Methylobacterium, and Rhizobium*. These genera have been well known for their potential as biocontrol agents and for enhancing plant health. This indicates a successful shift in the community structure from a pathogenic community to a relatively advantageous microbiome community after treatment with *P. parafulva* SAPEU-1.

##### 3.4.5.3 LEfSe cladogram of differentially enriched taxa

The Linear Discriminant Analysis Effect Size (LEfSe) identified the observed bacteria that were significantly enriched in the treatment. The red branches represent bacteria predominantly found in the pre-treatment samples, and the blue branches denote bacteria that increased post- *P. parafulva* SAPEU-1 inoculation. *Pseudomonas, Sphingomonas, and Methylobacterium* were identified as markers of the phyllosphere community in the treatment. *Xanthomonas* and *Microbacterium* markers were identified in the diseased control. The magnitude of the linear scores indicates that *Pseudomonas* is the most represented genus post-treatment. This identifies the successful colonization and establishment of the introduced endophyte.

##### 3.4.5.4 Differential abundance and student’s t-test at genus level

The quantification of relative abundance, carried out using the t-test (**Figure 6D**), showed a marked increase in *Pseudomonas* in the treated samples (p < 0.05) and a significant reduction in *Xanthomonas*. At the genus level, *Pseudomonas* and *Methylobacterium* showed moderate enrichment, whereas *Staphylococcus* and *Microbacterium* exhibited a marked reduction. From the Log2FC plot (**Figure 5D**), it is evident that the advantageous bacteria showed a marked positive enrichment. Conversely, those associated with pathogens had a marked negative enrichment. These results indicate a significant increase in beneficial bacteria and a corresponding decrease in those associated with pathogenesis.

#### 3.4.6 Species-level community profiles

##### 3.4.6.1 Network analysis before treatment

**Figure 7:**
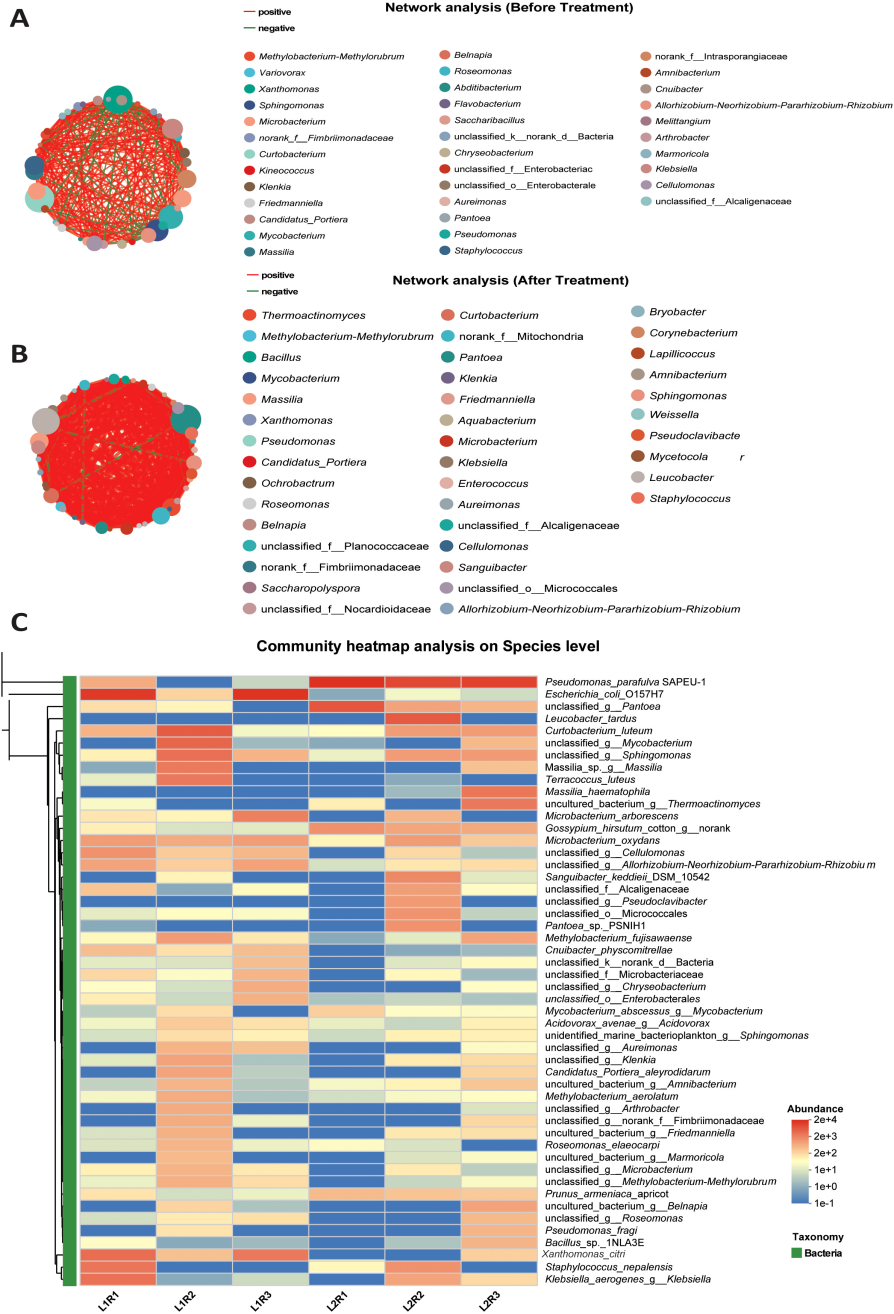
Microbial community structure at the Species-level of citrus phyllosphere microbiota before and after *Pseudomonas parafulva* SAPEU-01 treatment. **(A)** Network structure before treatment showing dense, pathogen-associated interactions dominated by *Xanthomonas*, *Microbacterium*, and *Staphylococcus*. **(B)** Post-treatment network showing increased positive associations and the formation of new hubs by *Pseudomonas*, *Bacillus*, and *Sphingomonas*. **(C)** Heatmap of species-level abundance, highlighting the decline of pathogenic taxa and the enrichment of beneficial species such as *Pseudomonas parafulva* SAPEU-01 and *Methylobacterium* after treatment.

The co-occurrence network of bacteria before *P.parafulva* SAPEU-1 inoculation (**Figure 7A**) exhibited a complex, dense connectivity pattern, suggesting dense microbial interactions in the diseased phyllosphere. This data showed both positive (red edges) and negative (green edges) associations between bacteria. This indicates the dynamic nature of interactions between bacteria, which can be either cooperative or competitive. The top hub species included *Methylobacterium–Methylorubrum, Xanthomonas, Microbacterium, Massilia*, and *Staphylococcus.* This community exhibited a tight structure with high association, suggesting its importance in maintaining the disease-causing microbiota. However, a strong association with competitive (negative) interactions is observed among genera associated with pathogens. This indicates instability in the community due to stress caused by canker. *Xanthomonas* showed high connectivity to other opportunistic bacteria. This means high dominance and possibly the repression of supportive bacteria.

##### 3.4.6.2 Network analysis after treatment

After treatment with *P. parafulva* SAPEU-1 (**Figure 7B**), the co-occurrence network evolved into a relatively balanced and mutually supportive form, characterized by a larger fraction of positive connections. A majority of the network’s constituents belonged to the non-pathogenic or commensal groups such as *Pseudomonas, Sphingomonas, Methylobacterium, Bacillus*, and *Rhizobium*. The appearance of network hubs for *Bacillus* and *Sphingomonas*, representing newly included plant-protective bacteria, indicates their introduction after inoculation with S2.

The connectivity between *Xanthomonas* and *Staphylococcus* improved slightly, although there is still less pathogenic dominance. The high connectivity among *Pseudomonas*, *Methylobacterium*, and *Bacillus* suggests the potential for synergistic interactions that could enhance microbial resistance and biological control in the phyllosphere. The post-treatment topological changes reflect a transition from a disease-driven competitive environment to a coordinated, biologically stable microbial community.

#### 3.4.7 Species-level heatmap analysis

In a species-level heat map analysis of phyllosphere microbiome data following treatment of diseased citrus plants with SAPEU-1, a phyllosphere isolate of *Pseudomonas parafulva*, the microbiome community structure underwent a marked alteration. Post-treatment, a highly significant enrichment of *P. parafulva* SAPEU-1 and *Pseudomonas fragi* was observed, indicating successful colonization of the introduced endophyte (**Figure 7C**).

It is pertinent to note that a highly significant decline in the counts of the pathogenic bacterium *Xcc* which causes citrus canker disease, was also observed following SAPEU-1 treatment. Alongside this, a marked decrease in the relative abundance of *Escherichia coli* O157:H7, a human pathogen that also acts as an opportunistic phyllosphere microorganism, and *Klebsiella aerogenes*, a human pathogen often found in opportunistic phyllosphere niches, was noted. Importantly, beneficial/neutral microorganisms in higher quantity—namely, *Leuconostoc tardus*, known for its antimicrobially harmful and stabilizing plant microbiome property, and *Sphingomonas*, known to associate with a healthy plant microbiome that stimulates overall nutrient cycling—were also found to have increased in quantity after SAPEU-1 treatment. Moreover, while a marked decline in overall phyllosphere opportunistic microbe *Escherichia coli* O157:H7 was observed after treatment, a marked increase in *Curtobacterium luteum*, a non-c-pathogenic phyllosphere microorganism that often colonizes healthy plant phyllospheres, was noted. Collectively, these data support the conclusion that SAPEU-1 treatment not only suppressed the pathogen *Xanthomonas citri* but also induced microbiome re-engineering, reducing opportunistic taxa and promoting beneficial assemblages, thereby enhancing phyllosphere stability and likely contributing to disease resilience.

## 4 Discussion

### 4.1 Biocontrol through both direct antagonism and microbiome engineering

Conventional biocontrol research tends to focus mainly on direct antagonism. This relates to the capacity of a biocontrol strain to inhibit pathogen growth through antibiotic production or lytic enzyme production (Haas and Défago, 2005; Mercado-Blanco and Bakker, 2007). This paper aims to demonstrate that *P.parafulva* SAPEU-1 not only directly inhibits *Xanthomonas citri* but also causes community restructuring of the phyllosphere microbiota, thereby improving the overall balance of the microbial community and transitioning it from a diseased and impoverished state to a sound, healthy state. This can be attributed to both antagonism and modification of the microbiota. This is related to the relatively new field of microbiome engineering (Du et al., 2025).

Phylogenetic analysis placed SAPEU-1 within the *P. parafulva* lineage; however, the moderate bootstrap support (41–58%) likely reflects the limited resolution of partial 16S rRNA sequences for differentiating closely related *Pseudomonas* species. *P. parafulva* has been encountered earlier for its potential plant-benefiting traits, such as antagonism against fungal diseases and internal colonization (Uchino et al., 2001; Dutta et al., 2019). For soybeans, the *P. parafulva* JBCS1880 isolate has been tested as a biocontrol agent for bacterial pustule control. But biofilm production did not improve its biocontrol potential in this regard. Experiments had been conducted to examine its applicability to citrus foliage (Dutta et al., 2019; Villamizar et al., 2020).

### 4.2 Alterations in α-diversity of citrus phyllosphere communities

The bacterial diversity of the citrus phyllosphere decreased after *P. parafulva* SAPEU-1 treatment according to ACE and Chao1 metrics, while Simpson index values showed increased community evenness. The microbiome of citrus phyllospheres underwent specific changes instead of random microbial population decline. The observed patterns appear in successful biocontrol systems, which use beneficial bacteria to control susceptible or functionally equivalent species through their competition for resources and their ability to occupy ecological spaces and create antimicrobial substances that enable the maintenance of a stable core community structure (Hassani et al., 2018; Kassa et al., 2023). The concurrent decline in Shannon diversity suggests that the reduction in richness outweighed gains in evenness, highlighting richness as a major driver of diversity shifts in treated phyllosphere communities. The combination of microbiome simplification with increased evenness between microbial species leads to better ecological stability and improved resistance against pathogens, which mainly affects plant compartments above ground because their microbial interactions remain active (Coyte et al., 2015; Toju et al., 2018). The Good’s coverage values showed consistently high levels throughout the analysis, which suggests that these diversity changes occurred through natural biological processes instead of sequencing errors. The research findings demonstrate that SAPEU-1 functions as a pathogen antagonist that also transforms the phyllosphere microbial community into a stable equilibrium that supports disease control through biological means (Bashir et al., 2022; Sarver et al., 2025).

### 4.3 β-diversity separation: community convergence post treatment

In the PCoA plot, a clear distinction is evident between the pre- and post-treatment microbial communities, with the first two axes accounting for a substantial amount of variance (51.36% and 27.92%). This indicates that the treated samples are more densely clustered than the sick samples. This situation means that S2 treatment tends to converge microbial communities away from random variance and towards a similar community structure driven by the selective pressure exerted by S2. This is evident in the Bray-Curtis distance boxplot, which indicates decreased variance within groups and increased variance between groups. This means that there is a marked effect of systematic disruption of community structure rather than random disruption. This is indicative of deterministic community assemblies rather than random or neutral phenomena. A similar finding from investigations on plant microbiota is that biocontrol inocula have a selective pressure effect on the community composition of phyllosphere microbes, with similar effects on ordination (Yuan et al., 2024). The bacterium not only becomes incorporated into the community but also alters the community’s overall environment.

### 4.4 Taxonomic and compositional reconfiguration: beneficial takeover

#### 4.4.1 Phylum-level trends

The data indicated a rise in Proteobacteria dominance at the phylum level (from 66% to 73%) post-S2 treatment. This suggests that *Pseudomonas* or related Proteobacteria play a key role in the ecological niche. This is because Proteobacteria consistently dominate the phyllosphere microbiota, followed by Actinobacteria, then Firmicutes, and finally Bacteroidota (Bashir et al., 2022; Sarver et al., 2025); there is a notable decline in Actinobacteriota and a slight decrease in Firmicutes/Bacteroidota. This could be a consequence of competitive exclusion or resource diversion.

#### 4.4.2 Family-level shifts

Changes at the family level offer more information. Before treatment, families associated with diseases or opportunistic bacteria, such as Xanthomonadaceae, Staphylococcaceae, and Micrococcaceae, were prevalent. After treatment with S2, there was a marked increase in the Pseudomonadaceae and Sphingomonadaceae families, along with other families, including Comamonadaceae, Beijerinckiaceae, and Rhizobiaceae. The rise in Pseudomonadaceae families signifies the establishment of S2 bacteria and possibly co-congeners. The decline in Xanthomonadaceae is welcome, as it is likely to include *Xanthomonas citri*.

These changes can be related to the concept of “microbiome rescue,” wherein a non-native strain establishes a protective community that outcompetes undesirable lineages (Sarver et al., 2025). It has been found that a compositional shift of a similar magnitude also occurred in the leaf microbiome’s biological control of grapevine pathogens when beneficial inoculum was used (Rappo et al., 2025). One critical aspect to consider is that relative measures inherently possess a compositional aspect. This is because reducing one lineage can exacerbate others. Absolute quantification of model lineages using q-PCR can significantly enhance such analyses.

### 4.5 Genus and species-level dynamics: dominance, suppression, assembly

Analyses at the genus level and other visualizations, such as Circos figures, clusters, LEfSe analyses, and t tests/log fold change maps, suggest *Pseudomonas* takeover is prevalent in the treated samples. A consistent drop follows this in *Xanthomonas*, *Microbacterium*, *Staphylococcus*, and other genera. Cladograms obtained from LEfSe analyses reveal that *Pseudomonas*, *Sphingomonas*, *Methylobacterium*, and *Rhizobium* are indicators of healthy-treated samples. *Xanthomonas* is identified as the indicator for the diseased samples. The species-level heatmap indicates that pathogen-associated genera are predominant in the untreated samples. Beneficial species like *P. parafulva*, the *Sphingomonas* sp., the *Methylobacterium* sp., and the *Bacillus* sp. rank high to emerge as the topmost probable dominant post-treatment. This indicates that S2 not only successfully colonizes but also acts as a keystone species, shaping the community structure around it.

Previous studies have demonstrated similar behavior in pathogen-suppression strategies developed in other biocontrol agents, such as *Bacillus* and *Trichoderma* (Poveda et al., 2021). This is readily confirmed by the current network-level data. This indicates that the pre-treatment data had density and competition with *Xanthomonas* and *Microbacterium* as central hubs. After treatment, there is increased density with prevalent positive interactions. Modules evident during treatment revolve around *Pseudomonas*, *Methylobacterium*, *Bacillus* sp., and *Sphingomonas*. This can be seen as a means to ensure the efficacy of cooperative modules at lower pathogen densities. This transformation in topological data corresponds to the report, in accordance with disequilibrium transformation, of a transformed ecosystem with a pertinent restored microbial balance (Legein et al., 2020). Data at both the genus and species levels are connected to a coherent report. This signifies dominating behavior on both the genus and species levels. It behaves like a keystone in dominating pathogens. It draws benefactors. It ensures increased cooperative network moquestures.

### 4.6 Microbiome restructuring and pathogen suppression by endophyte *P. parafulva* SAPEU-1

The species-level heatmap outcomes indicate that treatment with *P. parafulva* SAPEU-1 resulted in profound shifts in the citrus phyllosphere microbiome. The sharp enrichment of *P. parafulva* and *Pseudomonas fragi* in treated samples provides strong evidence of successful colonization of the applied endophyte, while the concurrent and substantial decline in the relative abundance of *Xanthomonas citri* underscores the biocontrol efficacy of SAPEU-1. In addition, reductions in *Escherichia coli* O157:H7 and *Klebsiella aerogenes*—both recognized opportunistic phyllosphere colonizers—suggest that inoculation may suppress niche expansion by non-target opportunists, a phenomenon consistent with restoration of a healthier leaf ecosystem (Wang and Cernava, 2023; De Mandal and Jeon, 2023). Equally noteworthy is the enhanced presence of beneficial or neutral taxa such as *Leuconostoc tardus*, *Sphingomonas* spp., *Curtobacterium luteum*, and *Massilia* spp. treated samples; such taxa are increasingly associated with plant health and community stability in phyllosphere settings (Agbavor et al., 2022; Wang and Cernava, 2023). These shifts collectively indicate that SAPEU-1 functions not merely as a pathogen antagonist but as a keystone species driving the re-engineering of the phyllosphere microbiome, suppressing dominant pathogenic and opportunistic taxa while promoting a more diverse and resilient assemblage of beneficial microbes. This restructuring underscores the concept of phyllosphere microbiome modulation for plant protection—a concept gaining traction as the next frontier in foliar disease management (Yuan et al., 2024; Wang and Cernava, 2023).

### 4.7 Mechanistic inference: how might S2 orchestrate the shift?

#### 4.7.1 Antibiosis, siderophores, and nutrient competition

Antibiotic or secondary metabolism is another well-known biocontrol strategy in *Pseudomonas* bacteria (Gross and Loper, 2009; Mercado-Blanco and Bakker, 2007). Some *Pseudomonas* bacteria produce phenazines, 2,4-diacetylphloroglucinol, HCN, or cyclic lipopeptides (Oni et al., 2022). Although S2’s metabolism is not directly described in this investigation, *in vitro* antagonism evidence suggests the possible future use of one or more of them. Nutrient rivalry is a crucial yet often overlooked strategy. Investigations analyzing the influence of Pseudomonas putida JBC 17 on green mold revealed that the impairment of fungal spore germination was due to nutrient exhaustion rather than direct antagonism. S2 could monopolize scarce nutrients, such as iron or carbon, on leaf surfaces, thereby excluding *Xanthomonas* and opportunistic species. Siderophore-dependent iron chelation by *Pseudomonas* is well recorded as a rivalry strategy (Biessy and Filion, 2018). Under phylloxeric conditions, iron availability is less. Hence, producing superior chelators can ensure a competitive advantage.

#### 4.7.2 Induced systemic resistance and host-mediated effects

A second mechanism is the induction of systemic resistance (ISR), which is not mutually exclusive. A large number of Pseudomonas-based biocontrol agents induce ISR to prime the host’s immune response. This modulates the production of pathogenesis-related proteins, leading to the synthesis of secondary metabolites that create less pathogen-conducive, microbiologically selective environments (Pieterse et al., 2014; Bakker et al., 2007). It is postulated that S2 influences the phyllosphere’s physiology, including leaf surface chemistry, exudate composition, and antimicrobial metabolites, thereby promoting advantageous selectivity.

#### 4.7.3 Microbiome recruitment and coalescence

A successful colonization of S2 would provide ecological scaffolding, enabling the establishment of suitable commensal bacteria on the leaf surface, such as *Sphingomonas* and *Methylobacterium*. Once established, the introduced bacteria could contribute to pathogen control through cross-feeding, competitive exclusion, or mutualistic defense. The Coalescence effect is increasingly finding its place in the paradigm of Synthetic Community Design (Sarver et al., 2025).

### 4.8 Ecological feedback and stabilization

Once the beneficial taxa have been identified, they can contribute positively to community stability. A network with modules that exhibit features such as synergy, cross-protection, and metabolic complementarity can contribute to the increased stability of a microbiome, providing benefits against pathogen re-invasion. The transition to a cooperative structure from the competitive and unstable network suggests that the system is now in a basin with attractor-like properties (Kajihara and Hynson, 2024; Hernandez et al., 2021).

### 4.9 Placing this work in the broader context and limitations

Multiple studies validate the results. For instance, Poveda focuses on microbial biocontrol agents used to manage citrus bacterial diseases, emphasizing the importance of combined approaches and the community context (Poveda et al., 2021). The idea that the phyllosphere can be altered as a microbiome habitat remains essential today, as recent work uses novel or engineered consortia with the potential to suppress leaf pathogens (Legein et al., 2020). However, there is also a presentation of limitations on the part of the study. Relative measures drive the conclusion; therefore, without absolute quantification tools, such as q-PCR or direct cell counts, it cannot be confirmed or ruled out whether there is a corresponding absolute reduction in pathogenic lineages. Secondly, the study duration is one month; therefore, the stability of the microbiome community remains unknown. Finally, it is essential to note that laboratory or pot trials lack the associated real-world forces that are simulated simultaneously with experiments conducted in greenhouses or pots. Therefore, it is crucial to conduct field experiments. Additionally, the modulation of metabolic production and its contributions to ISR and transcriptomic alterations were not directly examined. Hence, another corresponding future assessment is defined.

### 4.10 Implications and future directions

This study provides evidence for the effectiveness and potential of a specific leaf endophyte as a vital biological regulator of leaf microbiota. The next crucial translation steps include: Verification of the alteration in absolute abundance with qPCR analyses of *Xanthomonas*, *Pseudomonas*, and relevant species. Metagenomic analyses, as well as other forms such as expression analyses and metabolic analyses, validate functional pathways, including antibiotic production, metabolism, and ISR activation. Time-series experiments validate successional phenomena and resilience to disturbance. Field experiments on different citrus varieties in diverse climates. Formulation development (for example, carriers, coatings, protectants) to improve survival rates, colonization, and efficacy. Construction of novel microbiome consortia with a focus on the S2 microbiome and related species through network co-occurrence and functional complementarity (Nunes et al., 2024; Sarver et al., 2025).

## 5 Conclusions

An indigenous endophytic bacterium, *Pseudomonas parafulva* SAPEU-1, has been reported to have potential biological control activity against *Xanthomonas citri* in citrus plants. Its mechanism involves direct antagonism and modulation of the associated phyllosphere microbiota. *P. parafulva* SAPEU-1 showed the highest inhibition percentage among the tested endophytic bacteria. Phylogenetic reconstruction confirmed its correct classification.

**Figure 8:**
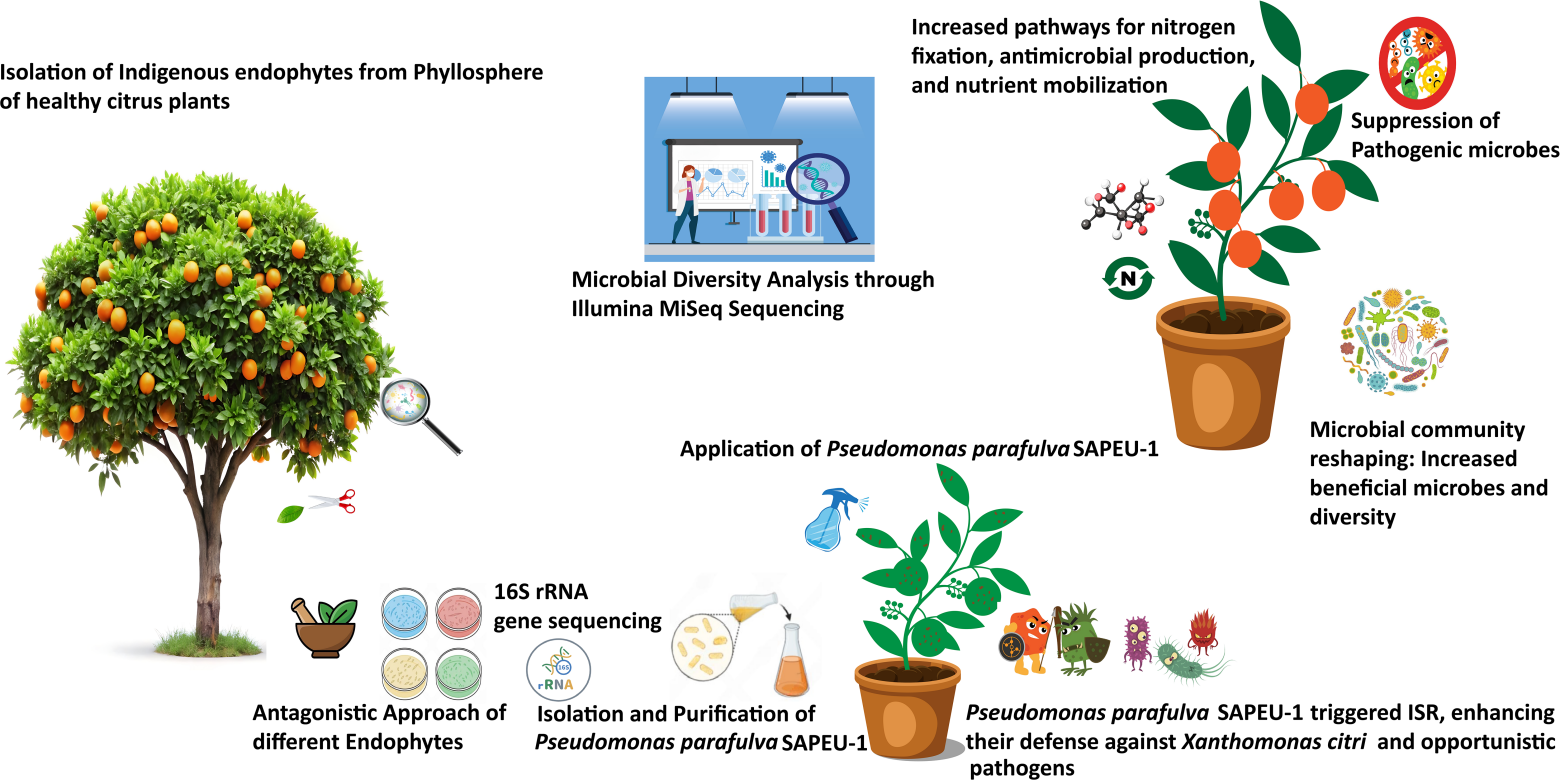
Concluding sketch of experiments performed in this study.

In pot trials with canker-contaminated citrus leaves, *P. parafulva* SAPEU-1 inoculation triggered a cascade of microbiome-level changes. These included increased α-diversity (OTU, Chao1, Shannon) and β-diversity (PCoA, Bray-Curtis distance), with a substantial shift in community structure. Specifically, post-inoculation samples formed a clearly separated and tightly grouped pattern compared to disease-control samples. S2 treatment led to the enrichment of OTU groups belonging to the Pseudomonadaceae, Sphingomonadaceae, and Rhizobiaceae families. This occurred concurrently with the attenuation of pathogen families, such as Xanthomonadaceae. *Pseudomonas* represented the vastly predominant genus and species post-inoculation. The co-occurrence pattern of modules transformed away from pathogen-related and competitive structures to more cooperative and stable ones. Functional reconstruction indicates potential enhancements to antimicrobial production pathways, nutrient metabolism pathways, or membrane transport (**Figure 8**).

## Data Availability

The datasets presented in this study can be found in online repositories. The names of the repository/repositories and accession number(s) can be found below: https://www.ncbi.nlm.nih.gov/, PQ895797 https://www.ncbi.nlm.nih.gov/, PRJNA1214773.
